# Prospective Evaluation of Procalcitonin, Soluble Triggering Receptor Expressed on Myeloid Cells-1 and C-Reactive Protein in Febrile Patients with Autoimmune Diseases

**DOI:** 10.1371/journal.pone.0153938

**Published:** 2016-04-20

**Authors:** Chou-Han Lin, Song-Chou Hsieh, Li-Ta Keng, Ho-Sheng Lee, Hou-Tai Chang, Wei-Yu Liao, Chao-Chi Ho, Chong-Jen Yu

**Affiliations:** 1 Department of Internal Medicine, Far Eastern Memorial Hospital, New Taipei city, Taiwan; 2 Department of Internal Medicine, National Taiwan University Hospital, Taipei, Taiwan; 3 Department of Internal Medicine, National Taiwan University Hospital, Hsin-Chu Branch, Hsin-Chu, Taiwan; 4 Department of Internal Medicine, E-Da hospital, Kaoshiung, Taiwan; Morehouse School of Medicine, UNITED STATES

## Abstract

**Background:**

Both procalcitonin (PCT) and soluble triggering receptor expressed on myeloid cells-1 (sTREM-1) have been investigated separately as indicators of infection in patients with autoimmune diseases. Our study simultaneously evaluated both PCT and sTREM-1 along with C-reactive protein (CRP) in febrile patients with autoimmune diseases.

**Methods:**

Fifty-nine patients were enrolled in the study. The patients were categorized into the infection group (n = 24) or the disease flare group (n = 35). sTREM-1, PCT and CRP concentrations at fever onset were compared between the two groups of patients.

**Results:**

sTREM-1 and CRP did not differ between the two groups. PCT [median (range), ng/ml] was higher in the infection group than in the disease flare group [0.53 (0.02–12.85) vs. 0.12 (0.02–19.23), *p* = 0.001]. The area under the receiver-operating characteristic (ROC) for diagnosis of infection was 0.75 for PCT (*p* = 0.001), 0.63 for CRP (*p* = 0.09) and 0.52 for sTREM-1 (*p =* 0.79). Using 0.2 ng/ml as the cutoff value for PCT, sensitivity was 0.75 and specificity was 0.77. Negative predictive values for PCT were 92%, 87% and 82% for a prevalence of infection of 20%, 30%, and 40%, respectively. Neither immunosuppressants nor biomodulators affected the level of the three biomarkers. However, in patients treated with corticosteroids, the levels of sTREM-1 and CRP were significantly decreased compared with the untreated patients.

**Conclusions:**

Setting PCT at a lower cutoff value could provide useful information on excluding infection in febrile patients with autoimmune diseases. The possible effect of corticosteroids on the level of sTREM-1 as an infection marker deserves further study.

## Introduction

The prevalence of autoimmune diseases has increased recently (7.6–9.4%) because of increasing awareness of the diseases, improved diagnostic tests, and the formulation of revised classification criteria [1–3]. In the developed world, which has a lower prevalence of infectious diseases, there has been a striking increase in the incidence of autoimmune disease, such as type 1 diabetes and multiple sclerosis [4]. With the advent of corticosteroids and immunosuppressants, rapidly fatal forms of autoimmune diseases can be brought to a low activity state. However, autoimmune diseases are still chronic systemic inflammatory diseases associated with significant morbidity and mortality. Infection is the leading cause of mortality throughout the autoimmune disease course, far more than cardiovascular complications, cancer, and disease activity per se [5–7]. However, in rheumatologic practice, determining the optimal approach to febrile patients with autoimmune diseases is still a challenge. The disturbed immune status due to active disease and a tendency toward infection caused by medications make accurate diagnosis difficult at first.

Apart from standard care, including physical examination and image studies, laboratory tests, such as white blood cell counts, erythrocyte sedimentation rate (ESR) and C-reactive protein (CRP), are not specific in differentiating disease flare or infection [8]. In the search for an ideal biomarker to aid in reaching a diagnosis, procalcitonin (PCT) has been regarded as promising in previous reports [8–15]. However, the performance of PCT has been disputed due to the limitation of the retrospective design, analysis of repeated serum samples, or uneven disease distribution in these early studies [16, 17]. There is still an unmet need to find a new biomarker for this group of patients.

The triggering receptor expressed on myeloid cells-1 is expressed on neutrophils and monocytes upon exposure to bacteria and fungi. The role of the soluble form of TREM-1 (sTREM-1) as a biomarker for infection has been proven in many different settings [18–20]. Studies on autoimmune diseases that regard sTREM-1 as a potential biomarker for this group of patients are sparse and conflicting [21–24]. The conclusions are also limited by the small patient numbers and blood sampling before antibiotics treatment in these previous studies. In addition, all the studies focused on one disease only, so whether the results can be generalized to other autoimmune diseases is unknown. From these inconsistent observations based on PCT or sTREM-1 alone, it is apparent that there is a need to evaluate the diagnostic value of PCT with sTREM-1 simultaneously for this population of patients. Thus, we conducted this study to investigate the role of PCT and sTREM-1 along with CRP in differentiating infection from disease flare prospectively in febrile patients with autoimmune diseases.

## Patients and Methods

### Study population

This study was approved by the Research Ethics Committee of the National Taiwan University Hospital. The study was performed at National Taiwan University Hospital. The institutional review board approved the study, and the patients or their relatives provided written informed consent. All patients 18 years of age or older who were hospitalized in our rheumatologic ward due to clinical exacerbation were screened for enrollment. Inclusion criteria were an autoimmune disease diagnosed by a rheumatologist and fever without preceding antibiotic treatment during admission. The patients’ medications for their autoimmune diseases were reviewed. The enrolled patients were defined as having corticosteroids use if the patients received more than physiologic doses of systemic corticosteroids (7.5 mg prednisolone or equivalent) for more than 3 weeks. Use of immunosuppressants, such as cyclophosphamide or methotrexate, and biomodulators, such as rituximab or etanercept, was also recorded.

Microbiological work-ups were performed on blood cultures, sputum cultures or specimens from other body regions that were suggestive of infection. Image study included chest radiography. Computed tomography or abdominal ultrasound was performed on an as-needed basis if the attending physician thought necessary.

The febrile patients were placed in the infection group if all of the following criteria were met: (1) detection of an infectious pathogen in culture with the physical or radiological findings compatible with an underlying infection (2) clinical response only to antibiotic treatment; no increased dose of corticosteroids or simultaneous adding of new immunosuppressants or biomodulators and (3) agreement between the attending physician and reviewing physician (C.C.Ho). The patients were diagnosed with a disease flare if there was (1) a negative microbiological culture, (2) fever responsive to increased doses of corticosteroids or adding up of new immunosuppressants or biomodulators, and (3) agreement between the attending physician and the reviewing physician. Patients who cannot fulfill the above three criteria or with concomitant infection and disease flare were excluded.

CRP was measured using the standard routine methods on an automatic analyzer (Hitachi). Additional samples were used for PCT and sTREM-1 analysis. PCT was determined by a commercially available homogeneous immunoassay using time resolved amplified cryptate emission (TRACE) technology (B.H.A.H.M.S. Diagnostica, Berlin, Germany). Plasma sTREM-1 concentration was determined by a sandwich enzymes-linked immunosorbent assay technique (ELISA).

### Statistical Analysis

Comparisons of CRP, sTREM-1 and PCT were analyzed using the Mann-Whitney *U*-test. Continuous variables were expressed as mean and standard deviation (±SD) or median (range) according to their homogeneity. Student’s t-test was used to compare mean values between two groups. Categorical variables were compared using the chi-square test or Fisher’s exact test. To evaluate whether the investigated biomarkers were able to reliably discriminate between patients with and without infections, receiver-operating characteristic (ROC) curves were generated. Statistical calculations were done with the SPSS software package (Statistical Program for the Social Sciences, version 16.0, Chicago, IL, USA),

## Results

### Patient characteristics

Fifty-nine patients were included in the study from 2009 to 2012, which included 24 in the infection group and 35 in the disease flare group. All 24 cases in the infection group had positive microbiological cultures. Systemic lupus erythematosus (SLE) was the most frequent underlying diagnosis in both groups (Tables 1 and 2). One patient with a disease flare resultant from Sjogren syndrome presented with fever, malaise and polyarthralgia. Her chest computed tomography disclosed areas of ground-glass attenuation. Her constitutional symptoms and fever responded well after elevating the dose of corticosteroids.

**Table 1:** Clinical characteristics of the two groups of febrile patients with autoimmune diseases.

	Flare (n = 35)	Infection (n = 24)	*P*
**Age (years)**	44.4±15.4	49.2±16.9	0.26
**Female**	26 (74.3%)	19 (79.2%)	0.67
**Underlying diagnosis**			
Systemic lupus erythematosus	15	8	
Sjogren syndrome	1	4	
Vasculitis	2	3	
Undifferentiated connective tissue disease	4	0	
Dermatomyositis	3	1	
Adult onset Still disease	3	0	
Seronegative arthritis	2	1	
Antiphospholipid antibody syndrome	1	2	
Systemic sclerosis	1	2	
Autoimmune thyroiditis	1	1	
Ulcerative colitis	1	0	
Sweet syndrome	1	0	
Rheumatoid arthritis	0	1	
Palindromic rheumatism	0	1	
**Use of medication**			
Corticosteroid	26 (74.3%)	17 (70.8%)	0.77
Immunosuppressant	18 (51.4%)	13 (54.2%)	0.84
Biomodulator	8 (22.9%)	6 (25%)	0.85

**Table 2:** Infectious diagnosis of the 24 patients.

Cause of fever	Number	Diagnostic method
**Bacteremia**	7	Blood culture
GNB	5	
GPC	2	
**UTI**	8	Urine culture
**Disseminated NTM**	2	Sputum culture with skin pathology
**Pneumonia**	4	Sputum culture with chest-x-ray
**Infectious diarrhea**	1	Stool culture
**Septic arthritis**	1	Culture from synovial fluid
**Septic shock**	1	Blood culture

GNB, Gram-negative bacillus; GPC, Gram-positive cocci; UTI, urinary tract infection; NTM, non-tuberculous mycobacteria.

### sTREM-1, PCT and CRP concentrations in the two groups

**Fig 1:**
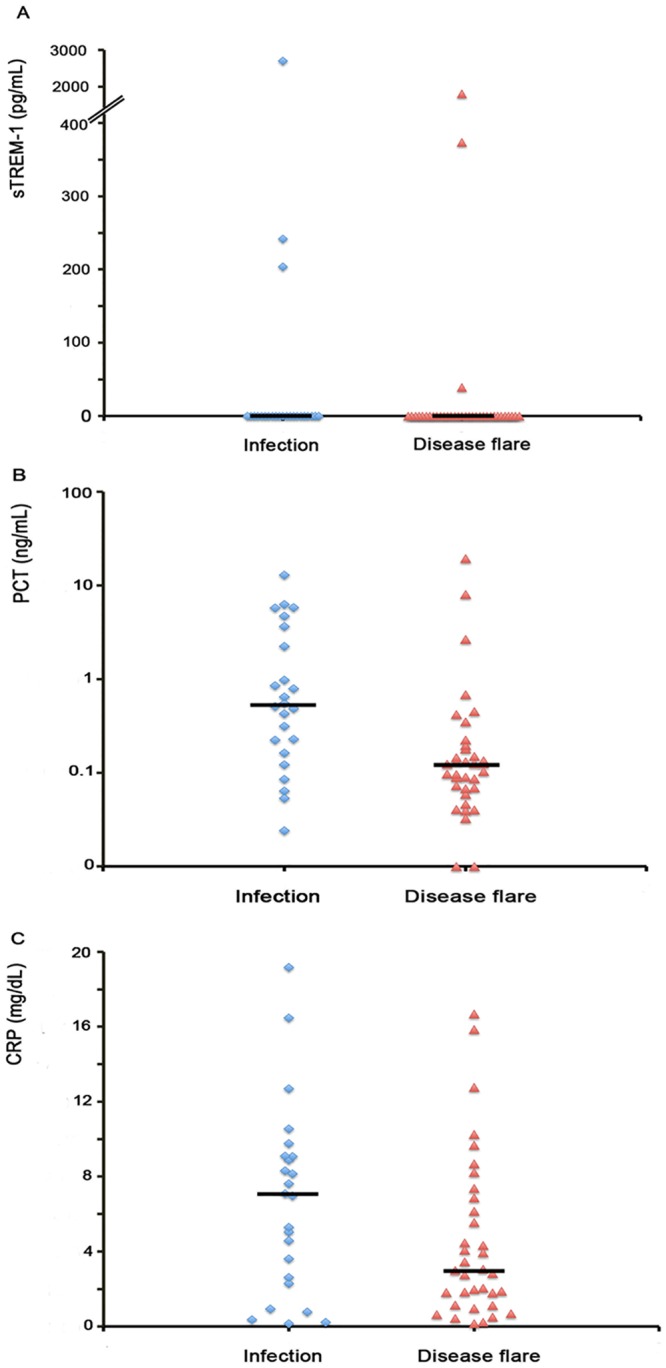
Individual plasma levels and medians of (A) soluble triggering receptor expressed on myeloid cells-1(sTREM-1); (B) procalcitonin (PCT); and (C) C-reactive protein (CRP) in the two groups. The horizontal bars represent the median values.

The median sTREM-1 in the infection group was not as elevated as in the disease flare group (median, 0 vs. 0; *p* = 0.62), while PCT was higher in the infection group than in the disease flare group (median, 0.53 vs. 0.12; *p* = 0.001). There was no significant difference in CRP level between the infection group and the disease flare group (median, 7 vs. 3; *p* = 0.09) (Fig 1).

**Fig 2:**
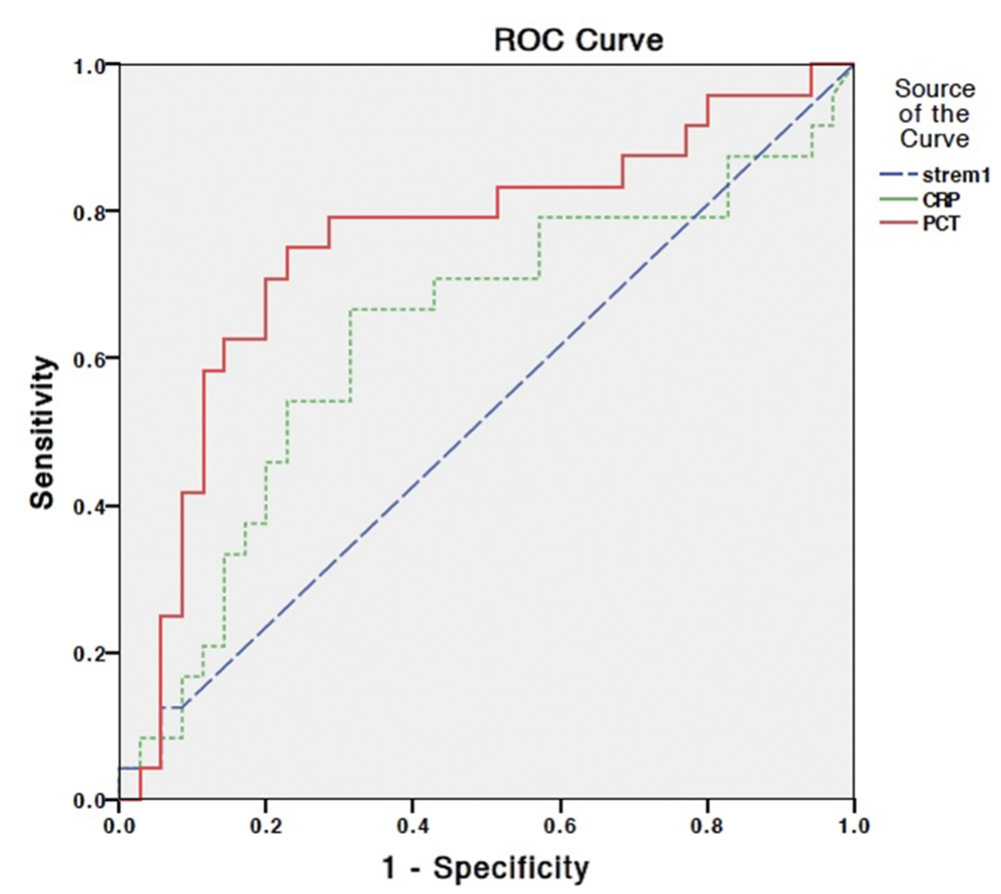
The receiver-operating characteristic (ROC) curves of soluble triggering receptor expressed on myeloid cells-1(sTREM-1), procalcitonin (PCT), and C-reactive protein (CRP) for prediction of infection. The area under the ROC curve was 0.52 for sTREM-1, 0.76 for PCT and 0.63 for CRP.

To evaluate the sensitivity and specificity of these three biomarkers, ROC curves were calculated (Fig 2). PCT was a good marker to discriminate the infection group from the disease flare group, with an area under the ROC curve (AUC) of 0.76 (95% confidence interval [CI]: 0.62–0.89; *p* = 0.001]. This is in contrast to sTREM-1 with an AUC of 0.52 (95% CI: 0.37–0.67; *p =* 0.79), and CRP with an AUC of 0.63 (95% CI: 0.48–0.78; *p* = 0.09). On the ROC curve, PCT level was combined the greatest sensitivity and comparatively good specificity, at 0.2 ng/ml (sensitivity: 0.75; specificity: 0.77). The negative predictive values were 92%, 87% and 82% for a prevalence of infection of 20%, 30%, and 40%, respectively.

### Effect of different therapeutic agents on sTREM-1, CRP and PCT

To evaluate the relationship between medications and biomarkers, the 59 patients were categorized into two groups based on whether therapeutic agents (immunosuppressants, biomodulators, and corticosteroids) were used. Neither immunosuppressants nor biomodulators lowered the level of sTREM-1, CRP and PCT (Table 3). In corticosteroid-treated patients, CRP was significantly decreased compared with untreated patients. We also found that patients treated with corticosteroids had significantly lower (mean rank = 27.6) levels of sTREM-1 than those without corticosteroids (mean rank = 36.4).

**Table 3:** Effects of immunosuppressants, biomodulators and corticosteroids on the level of the three biomarkers.

	Use of immunosuppressants		*P*	Use of biomodulators		*p*	Use of corticosteroids		*p*
	Yes (n = 31)	No (n-28)		Yes (n = 14)	No (n = 45)		Yes (n = 43)	No (n = 16)	
**sTREM-1 (IR), pg/ml**	0 (0–0)	0 (0–0)	0.27	0 (0–0)	0 (0–0)	0.61	0 (0–0)	0 (0–232)	0.001
**CRP (IR), mg/dL**	3.92 (2.04–8.22)	4.27 (0.75–8.83)	0.74	5.3 (2.72–9.23)	3.92 (0.94–7.89)	0.17	3.06 (0.95–6.97)	7.98 (2.7–9.6)	0.03
**PCT (IR), ng/ml**	0.12 (0.73–0.85)	0.17 (0.09–0.5)	0.55	0.38 (0.09–3.41)	0.14 (0.07–0.5)	0.18	0.13 (0.09–0.51)	0.18 (0.05–5.52)	0.53

sTREM-1, soluble triggering receptor expressed on myeloid cells-1; CRP, C-reactive protein; PCT, procalcitonin; IR, interquartile range.

## Discussion

According to our results, the sTREM-1 or CRP level alone on the day of a febrile episode in adults with rheumatologic disease was not useful in the differential diagnosis. PCT identified patients with infection with a relatively higher sensitivity and specificity.

Studies on the value of sTREM-1 in febrile patients with rheumatologic diseases were contradictory. Kim et al. and Hirayama et al. reported that sTREM-1 is capable of differentiating infections from active diseases [21, 23]. However, there are several aspects of the dataset that investigators should consider when applying the results. First, according to Kim et al. and Hirayama et al., the serial daily median level of sTREM-1 in the disease flare group was between 18.5 and 48 pg/ml. In the literature, the sTREM-1 in the group without infection was usually near zero [18–20], which differed from PCT or CRP. Moreover, there was also a potential for misclassification bias in the infection group, which comprised a larger proportion of clinically documented infection (bronchitis) cases or microbiologic cultures from non-sterile sites (pneumonia) [23]. Second, their results were limited due to the small patient numbers, with most of them receiving previous antibiotics treatment before blood sampling [21]. In our study, we categorized patients with microbiologically confirmed cultures, of which most were from sterile sites, into the infection group to avoid misclassification. As stated in our inclusion criteria, all the cases in the infection group were diagnosed with positive cultures and the patient’s clinical condition improved after sole antibiotic treatment without an increased dose of corticosteroids or adding up immunosuppressants or biomodulators. These strict inclusion criteria may help minimize bias. However, fourteen patients (58.3%) in the infection group did not have a systemic infection (8 urinary tract infection, 4 pneumonia 1 septic arthritis and 1 infectious diarrhea), and not all these cultures were from sterile sites (sputum culture and stool culture). There is concern that these patients with localized infection may have higher possibility to be misclassified. However, our results were valuable since the percentage (41.7%) of the patients with systemic infection in the infection group is the largest in the literature. The earlier two reports enrolled 11% (2/19; 1 disseminated tuberculosis and 1 bacteremia) and 0% of patients with systemic infection in their infection groups respectively [21, 23]. Our results are in line with the observation that either local sTREM-1 in the synovial fluid or serum sTREM-1 alone cannot discriminate infection or active disease [22, 24].

Recent studies showed that medication play an important role in the level of the biomarkers studied. Our results were in accordance with previous observations that CRP was attenuated by corticosteroids [25–27], whereas PCT was not altered [13, 25–29]. PCT also did not differ between the two groups in terms of immunosuppressant use, which was consistent with previous research [13, 30]. There have been no reports addressing the effect of biomodulators on biomarkers. Our study is the first to include biomodulators, which are an important component in the new treatment paradigm for rheumatologic patients.

The effect of corticosteroids on sTREM-1 in patients with infection has not been noted before. The level of sTREM-1 was very low in both the infection and disease flare groups. Our results reflected a recent animal study demonstrating that TREM-1 expression and sTREM-1 level were decreased after treatment with dexamethasone [31]. After the initial promising investigations of sTREM-1 as a biomarker for infection, its performance as a useful marker for infection is now debated [32, 33]. We believe that controlling the effect of corticosteroids in the study design of future research may help elucidate the role of sTREM-1 in the diagnosis or prognosis of infection.

In contrast to sTREM-1 or CRP, PCT had a relatively higher diagnostic value in febrile patients with autoimmune diseases. The sensitivity of PCT (0.75) is in accordance with a recent meta-analysis that also demonstrated a suboptimal result [34]. Since the inspiring results in patients with autoimmune diseases reported by Eberhard et al. [9], the performance of PCT in subsequent work has not always been satisfactory. This can be attributed to several factors. First, the infection groups in many of the previous reports were not precisely defined. Most of the infections were only diagnosed clinically [12, 14, 15]. Second, the control groups were heterogeneous, and some studies used systemic inflammatory response syndrome (SIRS) criteria rather than fever [11, 13–15]. Third, the long-term multiple therapies in patients with autoimmune diseases may modify PCT levels. Though corticosteroids were found to not affect the level of PCT significantly, PCT tended to decrease with higher doses and longer durations of corticosteroid use [26, 27, 29]. The use of corticosteroids was poorly described in these previous studies. Since our patients were chronically immunosuppressed, using the previously suggested 0.5 ng/ml of PCT as cutoff value will underestimate the numbers of patients with infection. We recommended setting a lower cutoff value of 0.2 ng/ml for this group in clinical practice. The reported prevalence of infection in patients with autoimmune diseases ranged mostly from 20% to 40% [34]. At a cutoff of 0.2 ng/ml, PCT levels can probably be used to predict the absence of infection in general, with negative predictive values ranging from 82% to 92%. Further trials should incorporate the PCT level as a treatment algorithm to see whether it can help reduce unnecessary antibiotic treatment.

Most of our study comprised of patients with diseases that characterized by a frequent relapsing-remitting course, such as SLE and vasculitis. However, some of the autoimmune diseases such as Sjogren syndrome typically have an indolent clinical picture and seldomly present systemic features. Another unique disease is systemic sclerosis hallmarked by its clinical heterogeneity that spans from mild cutaneous changes to an aggressive life-threatening multisystem disease. Our result is consistent with previous reports that only a few patients with flare of Sjogren syndrome or systemic sclerosis were reported [12, 15, 35]. Recognizing and quantifying these patients’ disease activity is much more difficult than it is for other autoimmune diseases. Like SLE and rheumatoid arthritis, an index for assessing disease activity was recently developed to help evaluate the patients with Sjogren syndrome or systemic sclerosis [36, 37]. More studies using these indexes to identify and describe these patients are warranted.

Despite our study’s strengths, there are several potential limitations that deserve consideration. First, we have a predetermined definition for the infection and the disease flare group unseen in previous similar studies. Under these objective criteria, patients categorized to the wrong group can be reduced. However, in the infection group, some of the patients were diagnosed with cultures from non-sterile sites (4 pneumonia and 1 infectious diarrhea) or with localized infection, and we could not exclude the possibility that some febrile patients with pathogens from colonization or contamination were categorized into the infection group. Despite the mentioned inclusion criteria, misclassification may have occurred due to diagnostic uncertainty, and this misclassification may have influenced the findings and results. Second, the enrollment criteria for infection group excluded patients with viral or mild localized infection. Our results have limited generalizability to clinical practice. Another limitation was that we did not measure the serial levels of these biomarkers after the initial blood sampling. Knowledge of these data can expand our knowledge of biomarker kinetics. Furthermore, the potential of being able to tailor therapy according to trends in the presence of biomarkers might permit more judicious use of antimicrobial agents.

In conclusion, the present results reveal that PCT can be an additional parameter for the differential diagnosis of febrile patients with autoimmune diseases. In the context of corresponding symptoms and clinical course, measurement of PCT may contribute to the exclusion of patients with infection. sTREM-1 or CRP cannot be used as a marker for the differential diagnosis between infection and disease flare. Our study, together with the results of previous studies, show that corticosteroids play an important role on the level of biomarkers, and this is the first study to raise the issue of the performance of sTREM-1 in patients receiving corticosteroids. The effect of corticosteroids should be taken into account in future studies and may at least partially explain the previous conflicting results concerning sTREM-1.

## Supporting Information

S1 Table All pathogens identified in the infection group. (DOCX)
