# Supplementary material for: Prospective Evaluation of Procalcitonin, Soluble Triggering Receptor Expressed on Myeloid Cells-1 and C-Reactive Protein in Febrile Patients with Autoimmune Diseases
Source: PLoS One. 2016 Apr 20;11(4):e0153938. doi: 10.1371/journal.pone.0153938 (PMC4838219; doi:10.1371/journal.pone.0153938)
Supplement: S1 Table — (DOCX) [file pone.0153938.s001.docx]

| Cause of fever |  | Pathogen Diagnostic method |
| --- | --- | --- |
| Bacteremia (7) |  | Blood culture |
| GNB (5) |  | *Pseudomonas aeruginosa* |
|  |  | *Pseudomonas aeruginosa* |
|  |  | *Enterobacter cloacae* |
|  |  | *Salmonella typhimurium* |
|  |  | *Salmonella typhimurium* |
| GPC (2) |  | *Staphylococcus aureus* |
|  |  | *Streptococcus viridans* |
| UTI (8) |  | *Escherichia coli* Urine culture |
|  |  | *Escherichia coli* |
|  |  | *Escherichia coli* |
|  |  | *Escherichia coli* |
|  |  | *Escherichia coli* |
|  |  | *Proteus mirabilis* |
|  |  | *Morganella morganii* |
|  |  | *Enterococcus* |
| Disseminated NTM (2) |  | M*ycobacterium kansasii* Sputum culture with skin  pathology |
|  |  | *Mycobacterium kansasii* |
| Pneumonia (4) |  | *Pseudomonas aeruginosa* Sputum culture with chest-x-ray |
|  |  | *Pseudomonas aeruginosa* |
|  |  | *Klebsiella pneumoniae* |
|  |  | *Staphylococcus aureus* |
| Infectious diarrhea (1) |  | *Salmonella*  Stool culture |
| Septic arthritis (1) |  | *Mycobacterium tuberculosis* Culture from synovial fluid |
| Septic shock (1) |  | *Escherichia coli* Blood culture |

**Table S1:** All pathogens identified in the infection group.

GNB, Gram-negative bacillus; GPC, Gram-positive cocci; UTI, urinary tract infection; NTM, non-tuberculous mycobacteria.
